# Neural Network Technologies for Age Estimation in Children from Orthopantomograms (a Pilot Study)

**DOI:** 10.17691/stm2026.18.2.02

**Published:** 2026-04-30

**Authors:** M.P. Poletaeva, Yu.V. Vasilevsky, D.K. Valetov, N. Angelakopoulos, G.V. Zolotenkova

**Affiliations:** MD, PhD, Associate Professor, Department of Forensic Medicine, N.V. Sklifosovsky Institute of Clinical Medicine; I.M. Sechenov First Moscow State Medical University (Sechenov University), 8/2 Trubetskaya St., Moscow, 119991, Russia; DSc, Professor, Corresponding Member of the Russian Academy of Sciences, Head of the Department of Higher Mathematics, Mechanics, and Mathematical Modeling, Institute of Computer Science and Mathematical Modeling, Biomedical Science and Technology Park; I.M. Sechenov First Moscow State Medical University (Sechenov University), 8/2 Trubetskaya St., Moscow, 119991, Russia; Assistant, Department of Higher Mathematics, Mechanics, and Mathematical Modeling, Institute of Computer Science and Mathematical Modeling, Biomedical Science and Technology Park; I.M. Sechenov First Moscow State Medical University (Sechenov University), 8/2 Trubetskaya St., Moscow, 119991, Russia; DDS, MSc, Department of Orthodontics and Dentofacial Orthopaedics; University of Bern, 7 Freiburgstrasse, Bern, 3010, Switzerland; MD, DSc, Professor, Department of Forensic Medicine, N.V. Sklifosovsky Institute of Clinical Medicine; I.M. Sechenov First Moscow State Medical University (Sechenov University), 8/2 Trubetskaya St., Moscow, 119991, Russia

**Keywords:** medical imaging, dental age estimation, orthopantomogram, dental status, deep learning, artificial neural network

## Abstract

**The aim of the study** was to investigate the potential of using artificial intelligence technologies for age estimation in children from dental radiographs.

**Materials and Methods:**

A retrospective study was conducted, analyzing orthopantomograms of 322 children (173 female, 149 male) aged 4–16 years. Fourteen permanent mandibular teeth were annotated on each radiograph. Neural network training was performed by splitting the data into training and test sets at a ratio of 80:20; 5-fold cross-validation was used. Age estimation was approached as a regression task. The neural network training and validation were conducted in Python using the PyTorch library. The accuracy of age prediction was assessed using the coefficient of determination (R^2^), mean squared error (MSE), and mean absolute error (MAE).

**Results:**

The study showed that the developed machine learning model was highly accurate in age estimation in children. The mean absolute error across cross-validation was 0.92 years, which was significantly lower than the error associated with traditional manual methods.

## Introduction

In the vast majority of cases, forensic medical expert practice relies on “manual” methods to measure and visually assess the dental status to estimate the age [1, 2]. The most popular among them are the following: the method of qualitative dentition estimation based on the stages developed by A. Demirjian; its modification proposed by G. Willems; and the quantitative method developed by R. Cameriere. The Demirjian’s method is based on determining the stages of tooth formation and development (eight stages from A to H) from a panoramic radiograph (orthopantomogram). The developmental stage of each tooth is converted into scores using a conversion table considering gender, and these scores are summed. The final maturity score is converted into dental age by using calculation tables or charts [3]. Willems subsequently slightly modified the method, simplifying the conversion of final scores into age, but the system of staging teeth according to the mineralization degree remained the method’s basis [3, 4]. Cameriere proposed a quantitative method for dental age estimation based on the correlation between age and the size of the open apices of tooth roots [5]. As practice has shown, these methods have a number of limitations. One of the main limitations is the requirement for the specialist to have at least a basic knowledge of dental development characteristics, understand the specifics of the method used, have enough time for the examination, and possess expertise in conducting this type of assessment. All these conditions affect the level of intra- and inter-expert consistency and can ultimately lead to inaccurate examination results [6–8].

Nowadays, the level of development of innovative technologies allows for the modernization of existing traditional methods. One option for increasing accuracy is the use of neural network technologies, namely machine learning methods. The application of digital innovations will make the age estimation faster, more accurate, and more objective [8]. Recently, the use of artificial intelligence has significantly expanded. The latest advances in deep learning in forensic medicine have shown promising results in age estimation using computed tomography scans of the knee joint [9], computed tomography scans of the neck [10], and other bone structures [11]. These studies demonstrate the great potential of using deep learning for age estimation. A number of studies in the field of artificial intelligence are devoted to the dental status assessment based on the analysis of third molars [6, 7], seven mandibular teeth [12], but the assessment of the entire mandibular dentition has not been carried out yet, which makes the scientific research up-to-date.

**The aim of the study** was to investigate the potential of using artificial intelligence technologies for age estimation in children from dental radiographs (orthopantomograms).

## Materials and Methods

### Sample characteristics

The study used panoramic dental radiographs obtained from the archival records of the Clinical Center of the Institute of Dentistry, I.M. Sechenov First Moscow State Medical University (Russia). The radiographs were taken for diagnostic and therapeutic purposes between 2017 and 2023. All images were obtained using a Pan eXam Plus digital panoramic X-ray machine (KaVo Dental, Germany) with the following exposure parameters: tube voltage — 66 kV, tube current — 2.5 mA, exposure time — 17 s. The files were saved in JPEG format with an original image resolution of 2800×1480 pixels.

**Figure 1.**
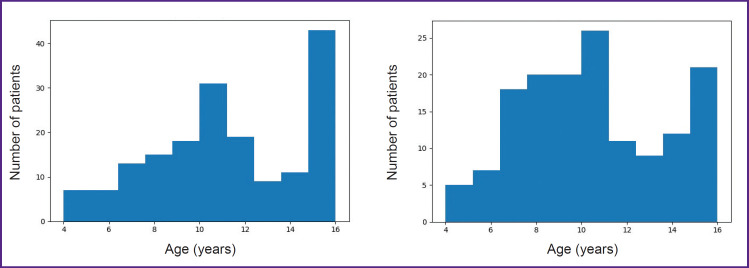
Distribution of the study sample by sex and age for female (left) and male (right) patients

A total of 322 orthopantomograms were included in the study. Exclusion criteria were the following: panoramic radiographs without information on patient sex, date of birth, or date of image acquisition; images from patients with a history of systemic diseases (including metabolic disorders and conditions affecting dental development); orthopantomograms showing visible bone tissue pathology; and poor-quality images. Patient age was calculated as the difference between the date of birth and the date of image acquisition. The age range was 4 to 16 years, with a mean age of 10.8 years. There were 46.3% males and 53.7% females. The distribution of patients by sex and age is shown in Figure 1.

The study was conducted in accordance with the Declaration of Helsinki (2024). The research protocol was approved by the Ethics Committee of I.M. Sechenov First Moscow State Medical University (protocol No.02-24, dated January 29, 2024). All data used in this study were anonymized to ensure confidentiality.

### Data preparation

**Figure 2.**
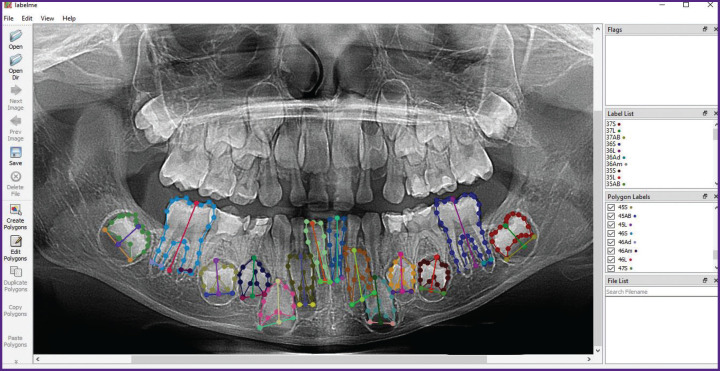
Example of annotation (labeling of tooth perimeter, tooth length, and root width) of permanent mandibular teeth using LabelMe software

In the initial stage, key parameters (sex and age) were extracted, and the orthopantomograms were simultaneously anonymized to prevent this information from influencing the model’s training process. Data annotation for machine learning was performed using the LabelMe software. On each orthopantomogram, the permanent mandibular teeth were annotated and labeled according to their corresponding tooth numbers (31–37 and 41–47), excluding the third molars (38 and 48). The following parameters were outlined for each tooth: tooth contour perimeter (S), metric characteristics including tooth length (L), and the width of the open apex/apices (AB, Ab, Ad). Depending on the stage of tooth development and mineralization, between two and four parameters were annotated per tooth. For example, for tooth 31, two parameters were annotated (perimeter — 31S; tooth length — 31L), while for tooth 36, four parameters were annotated (perimeter — 36S; tooth length — 36L; width of the distal and medial roots — 36Ad and 36Am, respectively). Figure 2 shows a fully annotated orthopantomogram image in the LabelMe software, where each parameter of the 14 teeth is outlined in a different color to ensure subsequent neural network training. The annotated file was saved in JSON format in a dedicated folder. The dataset was subsequently used for model training. The methodological basis for annotation was the original Cameriere’s method based on measuring the metric characteristics of seven left mandibular teeth. According to this method, a linear regression equation is used to estimate the child’s age [3].

### Neural networks

**Figure 3.**
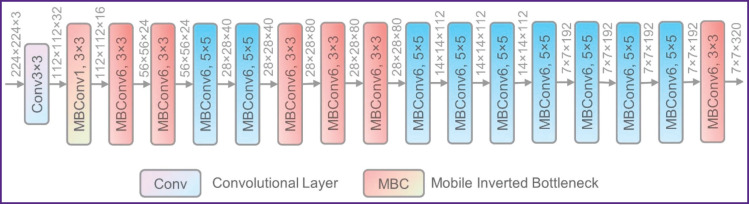
EfficientNet network architecture [15]

Machine learning methods, specifically neural networks, were used to further estimate the age. The age estimation task was approached as a regression in years using the EfficientNet-b0 neural network architecture [13–15] (Figure 3). This network was chosen due to its suitability with small datasets and positive results of its practical application. Neural network training and validation were conducted using the Python library PyTorch.

**Figure 4.**
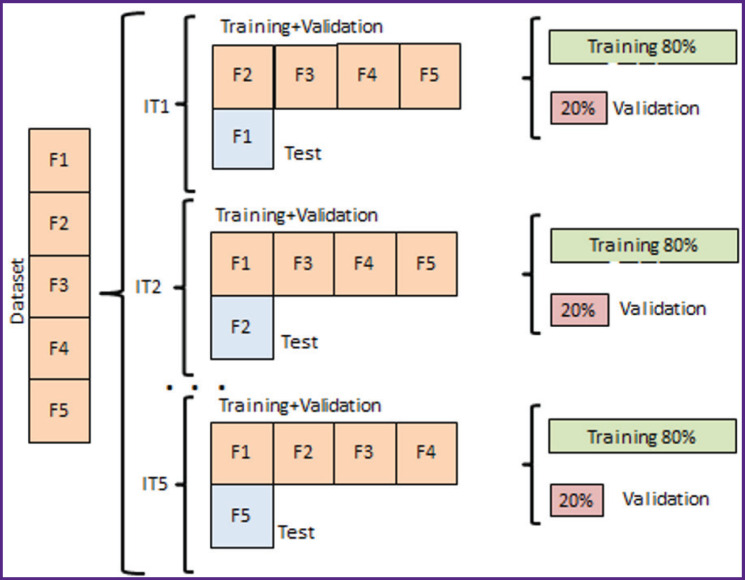
Cross-validation scheme used in the study (F1–F5 — sample subsets, IT1–IT5 — data iterations)

The regression-based age prediction task using the neural network was performed sequentially and consisted of the following stages. First, to reduce fluctuations in the neural network evaluation metrics, a 5-fold cross-validation of the model was conducted. The study evaluated the performance of both a single neural network and an ensemble consisting of varying numbers of networks of the same architecture, each trained from different random initial states. The general approach to model training and evaluation was the following: each model was trained for 50 epochs using 5-fold cross-validation. Training was performed by splitting the dataset in a 4:1 ratio, with 80% of the data used for training and 20% for testing. The system testing was conducted using a data block swapping method, where different data blocks were alternately used for testing against the training data to achieve the most stable mean absolute error (MAE) metric (Figure 4). During training, standard image augmentations (rotation, inversion, and mirroring) were used. The mean absolute error on the test dataset was evaluated at each epoch, and the best-performing model was saved. Optimization was conducted using the backpropagation algorithm, specifically the Adam (Adaptive Moment Estimation) method.

***Statistical analysis*** of the data included evaluation of age prediction accuracy using the following metrics: coefficient of determination (R^2^), mean absolute error (MAE), and mean squared error (MSE).

## Results

During the search for the optimal training parameters, the following values were identified and established: learning rate — 0.001, input image edge size — 224 pixels, and batch size for training — 16. To build the ensemble, independent sequential training of the models was performed using the Adam method. Table 1 shows the MAE values for different ensemble sizes obtained during testing and optimal model selection.

**T a b l e 1:** Table of values of mean absolute error (MAE) and mean squared error (MSE) in different numbers of neural networks

Error metric	Number of models								
	1	2	3	4	5	6	7	8	9
MAE±SD	1.014±0.066	0.949±0.080	0.931±0.089	0.927±0.093	0.927±0.093	0.936±0.103	0.927±0.103	0.920±0.099	0.920±0.101
MSE±SD	1.861±0.534	1.615±0.503	1.573±0.451	1.544±0.464	1.543±0.459	1.581±0.472	1.575±0.491	1.549±0.486	1.538±0.481

**Figure 5.**
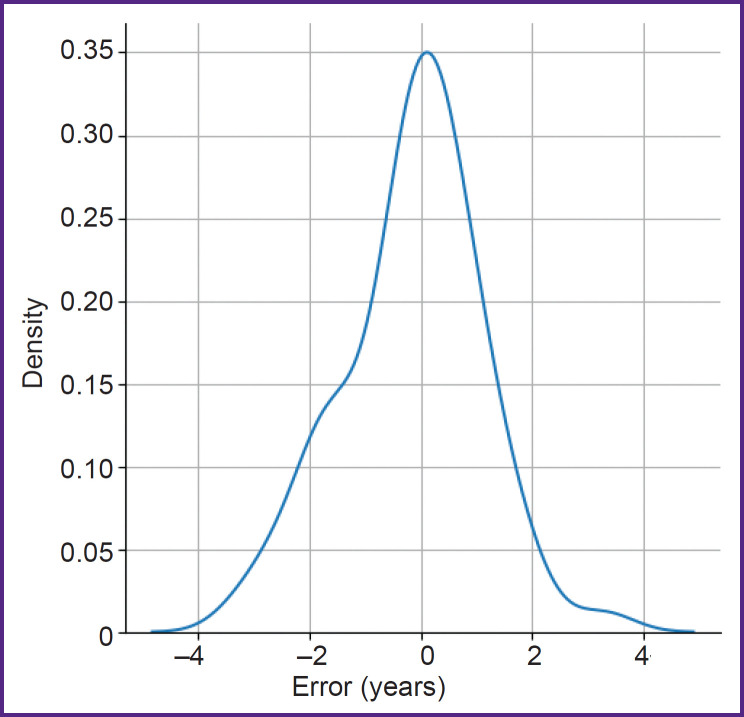
Error distribution graph for test data using an ensemble of five models

After that, the performance of the models within the ensemble was evaluated: predictions were obtained from each individual model on the test data and averaged with equal weights, which allowed to reduce prediction variance. Through cross-validation, the most optimal and acceptable training parameters were identified. Finally, an ensemble of five models was selected as the most accurate. When the ensemble size exceeded five models, the error margin did not further decrease, justifying the choice of this number. Thus, the mean absolute error from a single neural network model was 1.04 years, whereas in a case of the ensemble of five models it was 0.927 years (see Table 1). The error distribution for the five-model ensemble is demonstrated in Figure 5.

**Figure 6.**
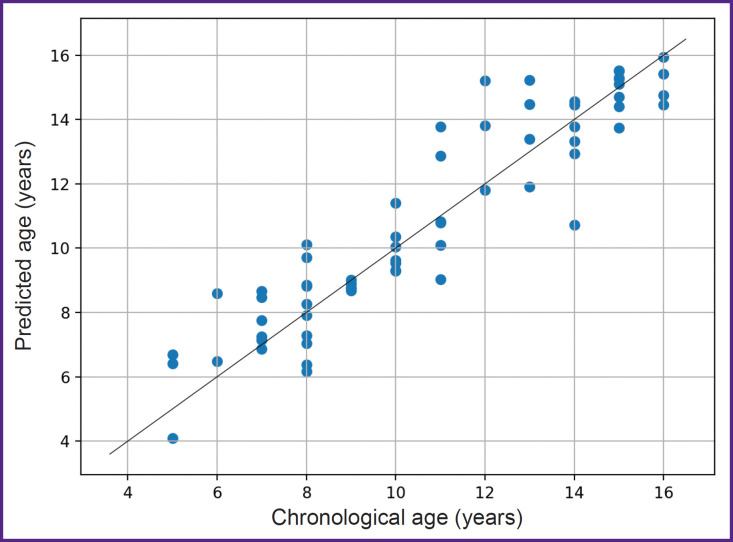
Joint distribution of chronological and predicted age using an ensemble of five neural network models on test data

The quality of the regression result was additionally visualized using a scatter plot of the test data based on predictions from the five-model ensemble, demonstrating an MAE of 0.92 and an R^2^ of 0.86 (Figure 6).

Analysis of the joint distribution of chronological age and predicted age showed that the majority of values fell within the desired value of the true age. Each point in the plot represents an individual sample from the test set, and its location (true age, predicted age) is close to the ideal position for accurate estimations. Therefore, the selected model successfully achieved an effective solution.

## Discussion

The majority of contemporary methods for dental age estimation involve a complex process of “manual” determination of a wide range of morphometric features, which are then compared to reference values stratified by sex and ethnicity, or involve measuring metric dental parameters followed by age calculation using regression equations. In international practice, the Demirjian’s and Cameriere’s methods are most commonly used for age estimation [3–5]. These methods have gained widespread acceptance across different countries due to their ease of use and acceptable results. However, “manual” methods have several limitations. The main one is connected with estimation difficulties caused by the task monotony and expert’s eyestrain, as well as lack of experience in analyzing radiographic images (orthopantomograms) and unfamiliarity/non-compliance with the estimation methodology [6]. Inter-expert variability in evaluating tooth development stages has also been noted, due to the common similarities between the adjacent mineralization stages. It may lead to overestimation or underestimation of chronological age [12, 16]. Consequently, increasing attention is paid to the development of automated algorithms (programs, methods) which can predict the age without expert involvement, therefore, eliminating subjectivity and improving prediction accuracy.

Research findings have demonstrated that, compared to original “manual” methods, all types of machine learning have greater accuracy [6]; however, error values vary depending on the underlying method (Demirjian, Cameriere, or Willems) used as the basis for annotation and on the machine learning approach employed (linear regression, random forest, support vector machines, etc). For instance, in a study by Galibourg et al. [17], a machine learning algorithm based on the Demirjian’s classification of radiographic stages of permanent teeth resulted in an MAE of 0.811 years, whereas “manual” estimation performed for comparison in the same study produced an MAE of 1.107 years. In the study by Shen et al. [12], the MAE for the traditional European Cameriere’s formula was 0.846 years, while machine learning methods based on Cameriere’s tooth maturation stages proved to be more accurate in estimating dental age: linear regression achieved an MAE of 0.55 years, and the support vector machine method had an MAE of 0.49 years. In the work of Tao et al. [18], assessment using the Demirjian’s method showed an MAE of 1.307 years for the entire sample, whereas error calculations using the MLP (multilayer perceptron) method were 0.75 years for the entire sample. In a study by Abuabara et al. [19], the accuracy of eight artificial intelligence models was compared with that of the traditional method. The results demonstrated that gradient boosting and random forest models had the highest performance and the lowest mean error. Thus, all studies confirmed the hypothesis of more accurate age prediction using machine learning methods (Table 2), which is consistent with the findings of the present study.

It should be noted that automated approaches using deep learning methods encounter a number of challenges in their planning and implementation. They also have some limitations, such as shortage of digital data, the time-consuming data annotation, selection of the optimal training method, and difficulties in fine-tuning, which influence the development and practical implementation of new methods [6, 12, 19]. This requires researchers to carefully select material appropriate for the study, ensure its compliance with inclusion criteria, develop a precise research plan and methodology, use reliable training methods, and engage qualified specialists with expertise in artificial intelligence.

**T a b l e 2:** Comparison of accuracy between traditional methods for age estimation in children from orthopantomograms (Demirjian’s and Cameriere’s) and various machine learning methods (according to the references)

Source	MAE value (original method)	MAE value (machine learning method)	Machine learning methods
***Demirjian’s method***			
[17]	1.107	0.811	Bayesian linear regression Decision tree Random forest
[18]	1.307	0.990	Multilayer perceptron
[19]	1.34	0.75	Linear regression Gradient boosting Support vector machine Multilayer perceptron Decision tree Random forest
***Cameriere’s Method***			
[12]	0.846	0.489–0.553	Linear regression Random forest Support vector machine

N o t e: MAE — mean absolute error.

The present study focused only on a sample of orthopantomograms from children aged 4 to 16 years; future research and results will be improved by modifying the sample characteristics through increasing its size and expanding the age range. In the following research, we will include materials from different centers and cover other age groups to create a more comprehensive dataset with a relatively balanced distribution of demographic characteristics. The ultimate goal of this project is to develop a new software product for age estimation in Russia with its subsequent practical implementation. It should be mentioned that similar tools are available at medical institutions in other countries; however, this is an innovation for Russia. Conducting research in this area corresponds to the healthcare digitalization, which is reflected in the development and implementation of modern information technologies leading to practical results being software creation.

## Conclusion

A machine learning algorithm for age estimation based on dental radiographs in children aged 4 to 16 years was presented and tested. By analyzing 14 permanent mandibular teeth and using a new set of data annotation features, with the help of linear regression and neural network methods, it was possible to successfully predict children’s age from orthopantomograms with a mean error of 0.92 years. These findings confirm the potential and real effectiveness of practical implementation of machine learning algorithms alongside with or instead of standard “manual” methods using assessment tables. This pilot project (experiment) has confirmed that an artificial intelligence-based algorithm is more accurate than widely used traditional methods for dental age prediction. The positive obtained results open up new opportunities for further integration of this algorithm into the practical work of both forensic medicine specialists and doctors in other clinical fields. The development of this scientific direction implies a quantitative increase and diversity of the sample, the use of various types of neural network algorithms, and their combinations to successfully achieve the ultimate goal being the software creation to improve the accuracy of age prediction based on dental status.
